# Low-Rank Matrix Denoising Algorithm-Based MRI Image Feature for Therapeutic Effect Evaluation of NCRT on Rectal Cancer

**DOI:** 10.1155/2021/3080640

**Published:** 2021-11-29

**Authors:** Qin Hu, Jin Li, Jun Li

**Affiliations:** Department of Radiology, The Jingmen No. 1 People's Hospital, Jingmen 448000, Hubei, China

## Abstract

This study aimed to explore the therapeutic effects of neoadjuvant chemoradiotherapy (NCRT) on rectal cancer patients using the MRI based on low-rank matrix denoising algorithm, which was then compared with the postoperative pathological examination to evaluate its application value in tumor staging after NCRT treatment. 15 patients with rectal cancer who met the requirements of radiotherapy and chemotherapy after conventional MRI were selected as the research subjects. The conventional MRI images before and after NCRT treatment were divided in two groups. One group was not processed and set as the conventional group; the other group was processed with low-rank matrix denoising algorithm and set as the optimized group. The two groups of images were observed for the changes in the ADC value and length and thickness of the tumor before and after NCRT treatment. The two groups were compared with the pathological examination for the complete remission of pathology (pCR) after the NCRT treatment and the tumor stage results. The results showed that Root Mean Square Error (RMSE) and Peak Signal to Noise Ratio (PSNR) (18.9121 and 74.9911 dB) after introducing the low-rank matrix denoising algorithm were significantly better than those before (20.1234 and 70.1234 dB) (*P* < 0.05); there were notable differences in the tumor index data within the two groups before and after NCRT treatment (*P* < 0.05), indicating that the NCRT treatment was effective. The pathological examination results of pCR data of the two groups were not much different (*P* > 0.05); the examination results between the two groups were different, but no notable difference was noted (*P* < 0.05); in the optimized group, there was no notable difference between the MRI results and the pathological examination results (*P* < 0.05), while in the conventional group, there were notable differences in the MRI results and pathological examination results (*P* < 0.05). In conclusion, MRI images based on low-rank matrix denoising algorithm are clearer, which can improve the diagnosis rate of patients and better display the changes of the microenvironment after NCRT treatment. It also indicates that NCRT treatment has significant clinical effects in the treatment of rectal cancer patients, which is worth promoting.

## 1. Introduction

Nowadays, people's living standards have been greatly improved resulting from the development of science and technology. Various aspects of clothing, food, housing, and transportation have seen changes of varying degrees, especially the food. People's lack of self-control in diet leads to the digestive tract problems. Colorectal cancer has been a relatively common gastrointestinal tumor China in recent years, and its incidence is increasing year by year. Rectal cancer accounts for nearly 70%, with the patients being younger [1]. According to the data of the International Cancer Research Center of the World Health Organization, in 2015, the number of people who died of cancer was as high as 8.8 million, among which 774,000 cases died of colorectal cancer, ranking the third among malignant tumors [2].

For the time being, the main method to treat rectal cancer is a combination of surgery with radiotherapy and chemotherapy [3]. If surgical therapy is used alone, there is a high probability of recurrence. For some advanced patients, the treatment effects are poor, and there is a high probability of recurrence and metastasis in a short time [4]. The surgery treatment hurts vitality, and the use of radiotherapy and chemotherapy is clinically controversial. In this regard, the National Comprehensive Cancer Network (NCCN) [5] guidelines identified NCRT as the preoperative radiotherapy and chemotherapy of patients to facilitate the surgery through tumor downgrading and tumor retraction [6]. Therefore, to evaluate the effects of NCRT in the treatment of rectal cancer has been a hot spot worldwide, with MRI being the main imaging detection method. MRI exhibits good resolution for soft tissues. It is safe and has no radiation damage [7]. Conventional MRI imaging can show the degree, size, and invasion of tumors, which can be observed in the middle and late stages of the tumor. It is the basis for preoperative staging [8]. Preoperative MRI can confirm the connection between the tumor edge and the mesangial fascia (MRF). This connection is an important anatomical basis to perform TME. MRI has high accuracy in judging whether the circumferential margin is positive, and the recurrence is closely related to the location of the lesion. To observe the angle between CRM and anorectum through MRI is conducive to adjusting the treatment plan and predicting the prognosis of the disease. However, in patients benefiting from NCRT, conventional MRI images cannot fully show the microenvironment of tumor cells when they die, limiting the therapeutic effect evaluation of NCRT [9]. This may be related to the noise pollution during the MRI imaging process or transmission process. Image noise will affect the subsequent MRI image processing and reduce the accuracy of diagnosis [10]. The details in the MRI image contain important medical information. The MRI image based on the low-rank matrix denoising algorithm can retain more effective medical information while restoring the image [11].

In the study, the MRI images based on the low-rank matrix denoising algorithm were used to detect the lesion after patients underwent NCRT, and the detection results were compared with postoperative pathological examination results to evaluate the application value of the optimized MRI in the detection of tumor changes after treatment and preoperative tumor staging. It was expected that the study can provide guidance for the detection and treatment of rectal cancer.

## 2. Materials and Methods

### 2.1. Research Subjects and Grouping

A total of 15 patients with rectal cancer admitted to our hospital from February 2019 to March 2020 were selected as the research subjects. Among them, 9 were male patients and 6 were female patients. They were between 18 and 67 years of age, with an average age of 49. After tested by conventional MRI, they all met the requirements of radiotherapy and chemotherapy. The conventional MRI images of all patients before and after NCRT were retained in two groups. One group was set as the conventional group without any processing; the other group was processed with a low-rank matrix denoising algorithm and set as the optimized group. The study was approved by the Medical Ethics Committee, and the patient had signed an informed consent form.

Inclusion criteria: (I) no previous malignant tumor treatment history; age > 18 years; (II) all had indications for preoperative radiotherapy and chemotherapy and can complete the treatment as planned; (III) able to complete MRI examinations at various time points as required; and (IV) surgical treatment and postoperative pathological staging four to eight weeks after NCRT treatment.

Exclusion criteria: (I) patients with rectal cancer who had contraindications to MRI examination; (II) patients who failed to complete NCRT as planned; (III) rectal cancer patients with incomplete images or poor image quality; and (IV) patients with severe heart, liver, and kidney function.

### 2.2. MRI Image Model Based on Low-Rank Matrix Denoising Algorithm

The adaptive median filter algorithm is used to preprocess the noisy data. Let the original MRI image be *F*={*f*_*k*_}_*k*=1_^*k*^, the noise-free image be *m*_*k*_, and the noisy image be *n*_*k*_.

(1) $$\begin{array}{c} f_{k}=m_{k}+n_{k}. \end{array}$$

There is a image block *p*_*ij*_ of an area of *m* × *m* with *j* as the pixel center point, and *z* approximate block {*p*_*i*,*j*,*k*_}_*i*=1_^*z*^ is found in the image *f*_*k*_, and the column vectors of the approximate block *p*_*i*,*j*,*k*_ are connected in series, *p*_*i*,*j*,*k*_ ∈ *R*^*n*^2^^, and a matrix *p*_*i*,*j*_ of *n*^2^ × *m* is defined as follows:

(2) $$\begin{array}{c} P_{j,k}=\left(P_{1,j,k},P_{2,j,k},\ldots ,P_{z,j,k}\right). \end{array}$$

Equation (1) can be expressed as follows:

(3) $$\begin{array}{c} P_{i,j}=M_{i,j}+N_{i,j}. \end{array}$$

**Figure 1:**
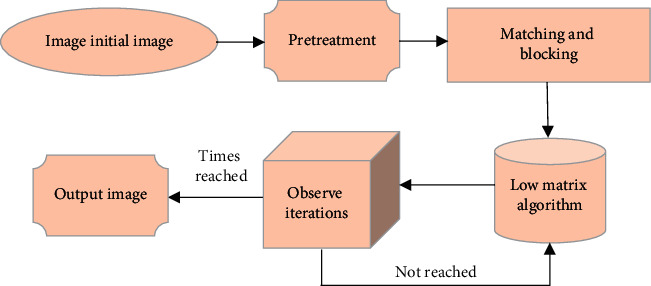
MRI processing.

The adaptive median filter algorithm [12] is used to preprocess the noisy data, and then, the corresponding matrix of the noise-free image is obtained through solving the minimization problem, and finally, the overlapping matrix blocks are merged to obtain a noise-free MRI image. The specific processing process is shown in Figure 1. The Root Mean Square Error (RMSE) and Peak Signal to Noise Ratio (PSNR) of the denoised image were then evaluated.

### 2.3. Treatment Plan

All patients accepted preoperative NCRT, and then, surgery was performed within 4 to 8 weeks. Neoadjuvant radiotherapy program: 45.0∼50.4 Gy/25∼28 f/5 w radiotherapy and short-term local radiotherapy. Neoadjuvant chemotherapy: 5-FU vs. capecitabine plus or minus oxaliplatin, respectively.

### 2.4. The MRI Examination

**Figure 2:**
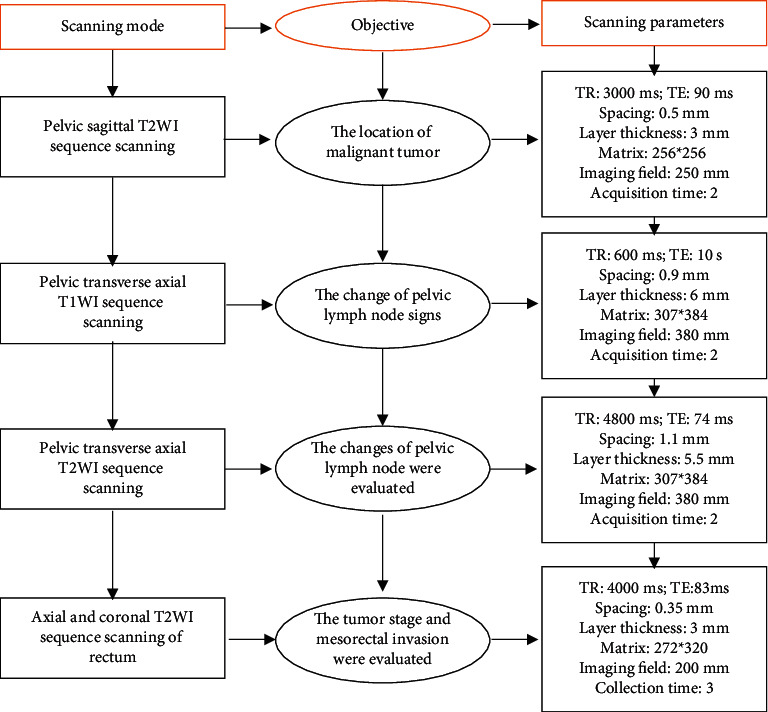
MRI scan process and relevant parameters.

- All patients were checked with a device of the same company. All digital and microscopic examinations in the intestines were prohibited within 12 hours before the examination. The patient should try to take chest breathing during the examination to reduce the interference to the MRI image for diagnosis.
- During the examination, the patient lied on the MRI examination table in a supine position to examine the whole pelvic cavity. The examination process is shown in Figure 2.

### 2.5. Evaluation Index

- Comparison of conventional MRI images and optimized ones.
- The change in the ADC value, length value, and thickness value of the tumor before and after NCRT treatment.
- Whether the pCR predicted by the conventional and optimized MRI images after NCRT treatment for rectal cancer was consistent with the results detected by the pathological examination after surgery.
- The changes in tumor staging (TNM staging method developed by the American Society of Oncology (AJCC) [13]) by conventional and optimized MRI images before and after NCRT treatment; whether NCRT treatment was effective for patients; and comparison of the results by pathological examination with those of the two groups. After NCRT treatment, the tumor staging of patients who were sensitive to NCRT may decrease; that is, the disease condition would get better. Therefore, only the staging results after treatment were compared.

### 2.6. Statistical Methods

All data were processed by SPSS22.0. The measurement data were expressed by *x*(−) ± *s*, and the *t* test was used. The count data were expressed as a percentage, and the *χ*^2^ test was used. *P* < 0.05 was the threshold for significance.

## 3. Results

### 3.1. Comparison of Denoising Effects

**Figure 3:**
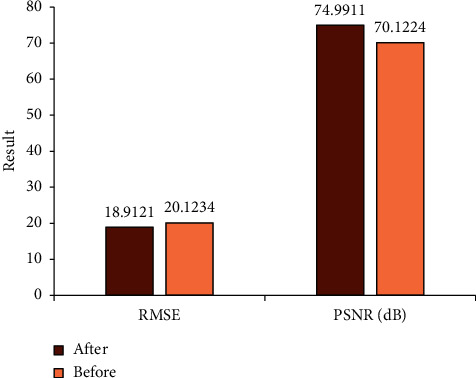
Comparison of the denoising effects.

**Figure 4:**
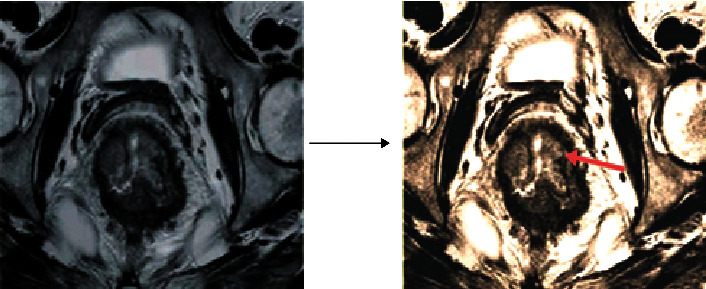
MRI images before and after low-rank matrix denoising processing (the red arrow indicated the lesion).

Figure 3 shows the RMSE and PSNR values of MRI images before and after introducing the low-rank matrix denoising algorithm. It was noted that RMSE and PSNR (18.9121 and 74.9911 dB) after introducing the low-rank matrix denoising algorithm were significantly better than those before (20.1234 and 70.1234 dB) (*P* < 0.05). The image processed by the low-rank matrix denoising algorithm was clearer than before, as shown in Figure 4.

### 3.2. The Patients' General Information

There was no notable difference in general data such as gender, age, and lesion location between the two groups (*P* > 0.05), and they were comparable, as shown in Table 1.

### 3.3. Comparison of the ADC Value, Length Value, and Thickness Value of the Tumor

I. The results of the ADC value, length, and thickness of the tumors before and after radiotherapy and chemotherapy are shown in Table 2.

According to the data of Table 2, the information below was obtained.

**Figure 5:**
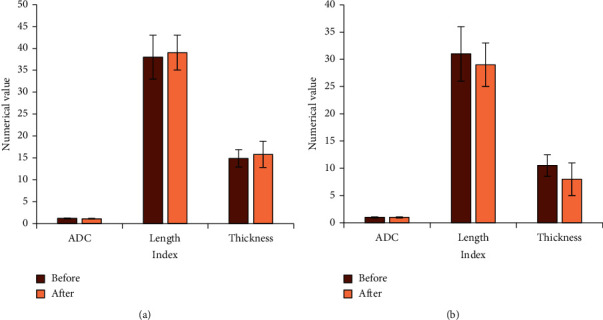
Comparison of tumor index data between groups. (a) Before treatment; (b) after treatment.

**Figure 6:**
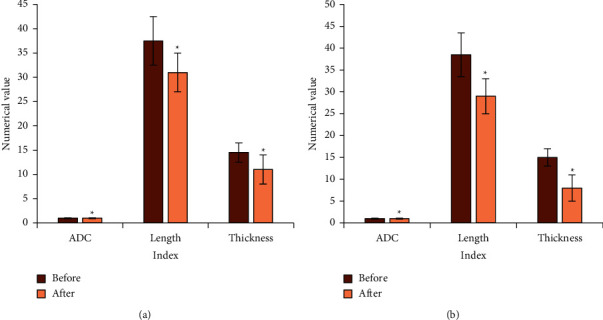
Comparison of tumor index data within groups. (a) Conventional group; (b) optimized group; “^*∗*^” means the comparison was statistically significant (*P* < 0.05).

- There was no notable difference in the tumor index data of the two groups of patients before NCRT treatment (*P* > 0.05) and after NCRT treatment (*P* > 0.05), as shown in Figure 5
- Notable differences were noted in tumor index data within the two groups before and after NCRT treatment (*P* < 0.05), indicating that NCRT treatment was effective, as shown in Figure 6

### 3.4. Comparison of pCR

**Figure 7:**
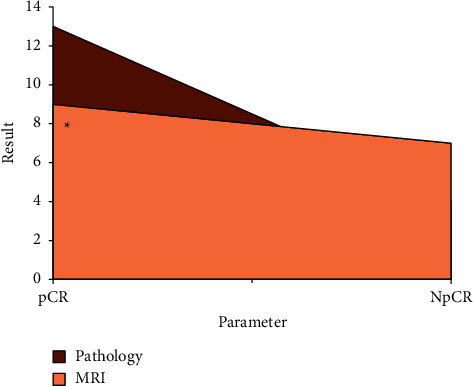
Comparison of pathological examination and conventional MRI examination. “^*∗*^” means the comparison was statistically significant (*P* < 0.05).

**Figure 8:**
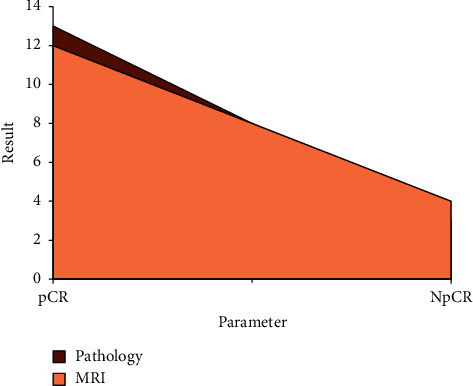
Comparison of pathological detection and optimized MRI detection.

As shown in Table 3, there was no significant difference between pCR data from pathological examination and the results from the optimized MRI (*P* > 0.05). The results by conventional MRI were different from those of optimized examination, but the difference was not notable (*P* > 0.05). In the optimized group, the pathological examination results were not much different from the MRI examination results, with no notable difference noted (*P* < 0.05), while in the conventional group, the pathological examination results were quite different from the MRI examination results, and the difference was notable (*P* < 0.05), which indicated that the MRI optimized by the low-rank matrix denoising algorithm can detect the pCR of the lesion more accurately, as shown in Figures 7 and 8.

### 3.5. Comparison of Tumor Staging Results

**Figure 9:**
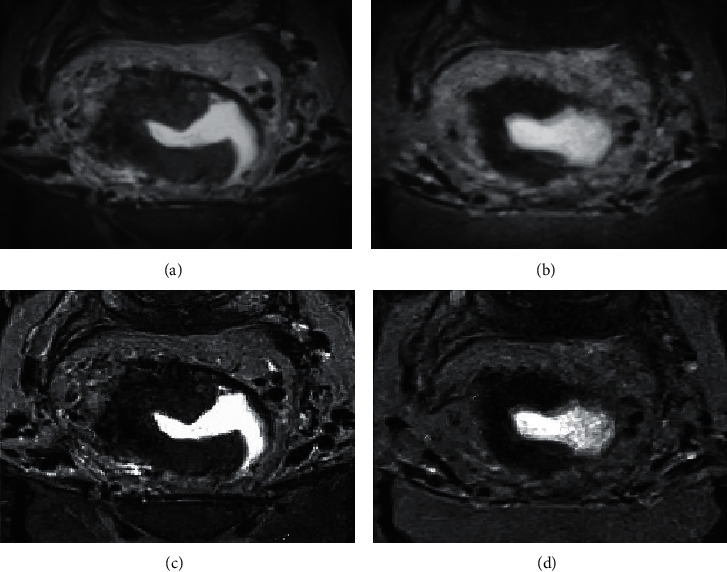
Comparison of conventional MRI and optimized MRI before and after NCRT. (a) Conventional MRI image before NCRT treatment; (b) conventional MRI image after NCRT treatment; (c) optimized MRI image before NCRT treatment; and (d) optimized MRI image after NCRT treatment.

**Figure 10:**
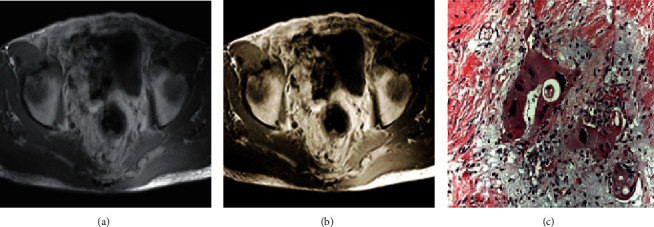
Comparison of conventional MRI, optimized MRI, and pathological results after NCRT. (a) Conventional MRI image diagnosed as TRG2; (b) optimized MRI image diagnosed as TRG2; and (c) pathological results diagnosed as TRG3.

The optimized MRI image was clearer, with smaller high signal range, but the contact range with the rectal wall was not obvious, and the stage cannot be defined, as shown in Figure 9. After NCRT, the staging results of conventional MRI were consistent with those of optimized MRI, but they were different from pathological examination results, as shown in Figure 10.

## 4. Discussion

According to the abovementioned result analysis, NCRT was effective in the preoperative down-stage treatment of rectal cancer. The conventional MRI can detect obvious changes in tumor morphology, while the pathological examination can detect the microenvironment changes inside the dead cells after NCRT. The image processed by the low-rank matrix denoising algorithm can restore the original information, displaying the morphological changes more clearly. The results showed that RMSE and PSNR (18.9121 and 74.9911 dB) after introducing the low-rank matrix denoising algorithm were significantly better than those before (20.1234 and 70.1234 dB) (*P* < 0.05). Thung et al. [14] also proposed that low-rank matrix denoising algorithm has a certain effect in processing MRI images. The results of this study were also consistent with the conclusions of Valvano et al. [15].

To have more accurate tumor staging after NCRT treatment, researchers have conducted a lot of exploration in this area. At present, MRI can clearly distinguish T1 and T2 stages, but it fails to distinguish tumors at the junction of T2 and T3 stages, easily causing misdiagnosis [16]. MRI demonstrates high tissue resolution, which can clearly show the penetration depth of the tumor on the rectal wall and the connection between the surrounding soft tissue and the tumor [17]. Sun et al. [18] studied the accuracy of high-field 3.0T-MRI in the diagnosis of rectal cancer and found that it had 100% sensitivity and 67% specificity in diagnosing muscularis propria invasion; that it had 91% sensitivity and 93% specificity in diagnosing surrounding tissue invasion; and that it had 64% sensitivity and 92% specificity in diagnosing lymph node metastasis. Zhang et al. [19] studied the accuracy of MRI in the circumferential margin detection before and after NCRT and found that the accuracy decreased after NCRT. It was because that the pathological tissue fibrosis occurred after NCRT treatment. Expert research shows that the accuracy of each stage after treatment is T stage- 50%, sensitivity- 100%, specificity- 35%, and N stage- 65% and accuracy of circumferential margin- 85% [20]. Subsequently, dynamic enhanced magnetic resonance technology was proposed, and studies suggested that MRI-enhanced scanning can improve the accuracy of rectal cancer staging [21]. Bakke et al. [22] found that the preoperative staging accuracy of dynamic enhanced MRI was as high as 92%. After NCRT treatment, tumor cells died, the cell morphology changed, and the production inflammatory substances led to cell edema, making it difficult to distinguish the boundary between the lesion and other tissue, so that the accuracy of T staging was reduced. Hence, improving the accuracy of MRI for tumor staging after NCRT treatment is very important. After all, the accuracy of diagnosis is closely related to the determination of the treatment plan and the therapeutic effects. Due to the continuous exploration and research of domestic and foreign researchers and the continuous improvement of MRI imaging technology in recent years, mature diffusion weighted imaging (DWI) technology has been widely used. The imaging principle of DWI is to detect the Brownian motion and the direction changes of water molecules, providing information about tumor pathological changes, vascular permeability, cell integrity, and water molecule dispersion movement, etc. The ADC value is a quantitative parameter that reflects the dispersion of water molecules within cells and tissue [23]. When using DWI to evaluate the therapeutic effects of NCRT, Zhang et al. [24] proposed that the ADC value after treatment increased first and then decreased. Caruso et al. [25] found that NCRT can affect the accuracy of ADC value in the diagnosis of rectal cancer. Oronsky et al. [26] studied the MRI data of 54 patients with advanced rectal cancer treated with NCRT and proposed that NCRT was effective in treating advanced patients, and indexes such as tumor ADC and difference change rate after treatment were instrumental in predicting the therapeutic effects. Taken together, it is evident that MRI is constantly being studied, improved, and then, applied to the clinic. Although there are still some problems, with the continuous development of technology, these problems will be overcome.

## 5. Conclusions

In the study, the MRI based on the low-rank matrix denoising algorithm was compared with conventional MRI and pathological examination to explore its application value in evaluating the therapeutic effects of NCRT for rectal cancer. The results showed that MRI images based on low-rank matrix denoising algorithm were clearer, which could improve the diagnosis rate of patients and better display the changes of microenvironment after NCRT treatment. It also suggested that NCRT treatment had significant clinical effects in the treatment of rectal cancer patients and was worth promoting. However, some limitations in the study should be noted. The sample size is small, which will reduce the power of the study. In the follow-up, an expanded sample size is necessary to strengthen the findings of the study. In conclusion, this study not only indicates that intelligent algorithm has a good development prospect in the medical field but also indicates that NCRT therapy should be widely used in cancer diseases.

## Figures and Tables

**Table 1:** General information of patients.

Gender	Number of people	Age (average)	Lesion		
			Upper	Middle	Lower
Male	9	45 ± 2.3	3	2	4
Female	6	53 ± 1.2	1	2	3

**Table 2:** The tumor index data.

Index	Conventional group		Optimized group	
	Before NCRT	After NCRT	Before NCRT	After NCRT
ADC value	0.95 ± 0.19	0.90 ± 0.09	0.95 ± 0.22	0.77 ± 0.05
Length	37.61 ± 9.72	30.21 ± 8.12	38.72 ± 7.51	29.21 ± 7.12
Thickness	14.01 ± 2.85	10.11 ± 3.10	15.05 ± 4.12	8.15 ± 2.41

**Table 3:** pCR data.

Parameter	Pathological examination	Conventional MRI examination	Optimized MRI examination
pCR	13	9^*∗*^	12^*∗*^
NpCR	2	6	3

*Note.* “^*∗*^” indicates that the comparison was statistically significant (*P* < 0.05), and NpCR indicates incomplete response.

## Data Availability

The data used to support the findings of this study are available from the corresponding author upon request.
